# A survey of testicular texture in canine ultrasound images

**DOI:** 10.3389/fvets.2023.1206916

**Published:** 2023-08-11

**Authors:** Fintan J. McEvoy, Panida Pongvittayanon, Tanja Vedel, Pernille Holst, Anna V. Müller

**Affiliations:** Department of Veterinary Clinical Sciences, Faculty of Health and Medical Sciences, University of Copenhagen, Frederiksberg, Denmark

**Keywords:** quantitative texture features, ultrasonography, quantitative image analysis, gray-level co-occurrence matrix, canine, testes

## Abstract

**Introduction:**

Computer-based texture analysis provides objective data that can be extracted from medical images, including ultrasound images. One popular methodology involves the generation of a gray-level co-occurrence matrix (GLCM) from the image, and from that matrix, texture fractures can be extracted.

**Methods:**

We performed texture analysis on 280 ultrasound testicular images obtained from 70 dogs and explored the resulting texture data, by means of principal component analysis (PCA).

**Results:**

Various abnormal lesions were identified subjectively in 35 of the 280 cropped images. In 16 images, pinpoint-to-small, well-defined, hyperechoic foci were identified without acoustic shadowing. These latter images were classified as having “microliths.” The remaining 19 images with other lesions and areas of non-homogeneous testicular parenchyma were classified as “other.” In the PCA scores plot, most of the images with lesions were clustered. These clustered images represented by those scores had higher values for the texture features entropy, dissimilarity, and contrast, and lower values for the angular second moment and energy in the first principal component. Other data relating to the dogs, including age and history of treatment for prostatomegaly or chemical castration, did not show clustering on the PCA.

**Discussion:**

This study illustrates that objective texture analysis in testicular ultrasound correlates to some of the visual features used in subjective interpretation and provides quantitative data for parameters that are highly subjective by human observer analysis. The study demonstrated a potential for texture analysis in prediction models in dogs with testicular abnormalities.

## 1. Introduction

The extraction of quantitative image features from medical images is an increasingly growing domain in human and non-human diagnostic imaging (1–3). Complex pattern recognition can be performed using advanced image analysis methods. These patterns can then be explored, compared, and potentially assist alongside subjective and operator-dependent quality assessment, image optimization, and image interpretation. This process is often referred to as radiomics (2, 4).

Diagnostic ultrasound is commonly used for examination of the testicles in dogs and it is a recommended part of routine abdominal ultrasound (5–7). Testicular ultrasound is excellent for the detection of palpable and non-palpable lesions in dogs that may be of neoplastic, inflammatory, cystic, or degenerative origin. Nodular lesions of different etiologies can however have similar ultrasonographic features (6, 8–10). Areas of testicular calcification can be seen ultrasonographically as hyperechoic pin-point foci in the testicular parenchyma, without acoustic shadowing. In humans, such findings are referred to as testicular microlithiasis (11, 12). Microlithiasis is not considered an independent risk factor but has been associated with several testicular conditions in men, including neoplasia and infertility (12, 13).

Subjective texture assessment of testicular ultrasound images has been used to investigate fertility in humans. An association between testicular appearances, such as homogeneity, irregularity, and semen quality in humans, has been reported in men (14, 15). Subjective scoring of testicular echogenicity and its potential correlation with fertility has been investigated by comparisons between a population of 10 dogs with established infertility and 10 healthy dogs. This study did not report a clear correlation between echogenicity and fertility (16). Objective texture features based on mean pixel intensity have also been investigated, with a reported negative correlation between objective testicular echogenicity and semen quality (5).

Texture analysis can be performed based on the spatial relationship among pixels. One approach is to consider two pixels at a time. That is a reference pixel and its neighbor. Various spatial relationships between the two are possible, the neighbor pixel could be one pixel to the right of the reference, or one to the left, or even one pixel diagonally to the right. The neighbor pixel may also be two or three pixels removed from the reference again in various spatial directions. Once the relationship is chosen, the frequency of each potential pixel value pairing in the image can be found. If for example the image is 8 bit there will be potential values from zero to 255 (i.e., 256 values). Potential pixel value pairings for a reference pixel value of zero will be (0,0), (0,1), (0,2)....(0,255): Similarly the number of occurrences of pairings with reference pixel of 1 can also be found. This process is continued until the frequency of all possible reference and neighbor pairings have been determined. The frequencies are recorded in a series of rows and columns called a gray-level co-occurrence matrix (GLCM). For an 8 bit image the matrix will have 256 rows and 256 columns. The number recorded in the cell at for example, row 100 column 50, will indicate the number of times in the image, the reference pixel value is 100 and neighbor pixel value is 50. From the GLCM, statistical features can be calculated and used for texture characterization. Examples of such features are energy, entropy, contrast, correlation, dissimilarity, homogeneity, and angular second moment (ASM) (17). Based on the calculation methods, a negative correlation can be expected between entropy and ASM, whereas a positive correlation is expected between dissimilarity and contrast (18). This type of texture analysis has a long history in image analysis and has been reported for a variety of applications, including medical ultrasound images of breast (19), liver (20), and muscle (21). Variations in GLCM texture features extracted from testicular ultrasound images have been reported to occur in association with abnormal testicular spermatogenic capability and as a result of the action of pituitary gonadotropins in men (22).

The aim of this study is to describe a quantitative, open-source method for extracting quantitative texture features from testicular ultrasound images from male dogs. The resulting texture features will be explored in light of available biological information about the dogs, such as age, body condition score (BCS), and visual findings in the analyzed images.

## 2. Materials and methods

### 2.1. Study population

Healthy male dogs were recruited prospectively for a focused testicular ultrasound examination. The dogs were recruited through the personal networks of the authors, and by sharing project flyers in veterinary clinics and dog parks in the Copenhagen area. Furthermore, recruitment posts were shared on the social media profiles (Facebook and Instagram) of the University Hospital for Companion Animals at the University of Copenhagen (UCPH-CA). The inclusion criteria were defined for a pilot study with another scope than the present study and therefore included intact males of 7 years of age or older without clinical history or clinical signs of testicular disease. A telephone interview with the owner was performed, and dogs were excluded if they had cryptorchidism, a history of either current or recent systemic disease, or signs of testicular neoplasia, such as alopecia or anemia. For the eligible dogs, the following data were recorded: age, breed, breeding status, known offspring, prior or current prostatic disease, and treatment with the GnRH agonist deslorelin acetate (Suprelorin, Virbac SA) or the antiandrogen osaterone acetate (Ypozane, Virbac SA). The dogs were then invited for an ultrasonographic testicular examination at UCPH-CA. Upon arrival, dog owners signed an informed consent form before the examination of their dogs. The weight and body condition score (23) were noted for each dog, and the testes were palpated for asymmetry or other abnormalities.

### 2.2. Ultrasonographic examination

All testicular ultrasonographic examinations took place in the period from February to November 2022, using a LogiQ E10 (GE Healthcare) ultrasound scanner with a 10–15 MHz linear transducer. All examinations were performed by one of the authors (AVM—DVM, Ph.D., assistant professor in veterinary diagnostic imaging). The dogs were awake and minimally restrained during the examination. Most dogs were scanned in dorsal or lateral recumbency, but a handful of dogs were scanned standing at the discretion of the dog and owner. For texture analysis, B-mode still images and left-to-right or cranial-to-caudal cine-loops of each testicle were acquired in mid-sagittal and mid-transverse planes. All images were saved in DICOM format in a picture archiving and communication system (PACS).

### 2.3. Image processing and feature extraction

A visual screening and subsequent selection of the saved DICOM images were performed. Two images of each testicle were chosen for texture analysis; a mid-sagittal image at the level of mediastinum testes and a mid-transverse image. Each image was exported from the PACS server (2019, PacsOne Server version 6.8.1) in a lossless imaging format [the portable network graphics format (png)]. These images were imported into a dedicated program running in the computer language Python (Version 3.7.2 Python Software Foundation, http://www.python.org). This program used the Scikit-image processing library (24), the library NumPy to provide support for large, multi-dimensional arrays and matrices, (25) and the plotting library, Matplotlib (26). The functions graycomatrix and graycoprops were imported from the skimage library to calculate the GLCM. Parameters for this function were set so that the GLCM data were normalized for each image, the output matrix was set to symmetric, paired distances were set to 5 pixels and the number of gray levels was set to 256 (8-bit). The program displayed the image, allowed hand selection of regions of interest, calculated a range of texture features, and exported these along with other image data to a comma-separated file (“csv” file). The features extracted were entropy, ASM, contrast, energy, dissimilarity, correlation, and homogeneity (17). Other image data stored included the image file name, the coordinates of the selected ROI, and user input to record the presence or absence of mineralization or nodules in the selected ROIs. The code is available as a “Jupyter Notebook” (27) and as an executable Python script together with a sample image at this Github repository Link.

### 2.4. Statistical analysis

The texture features derived from the GLCM were analyzed using principal component analysis (PCA). It is an unsupervised, qualitative, multivariate method that can be used to identify patterns in complex biological and image data sets and images (18, 28, 29). PCA identifies the main sources of variability in the given data and can therefore be used to identify similarities and dissimilarities between the analyzed features (30). In short, three types of information are given by PCA. One is a set of linear functions that successively maximize the variance and that are uncorrelated with each other. These functions are known as the Principal Components. When presented in a two-dimensional plot, the x- and y-axes represent the principal components. The PCA also creates a set of loadings that describe the covariance observed between the extracted texture features. A positive loading indicates that a texture feature contributes to some degree to the principal component, and a negative loading indicates that its absence contributes to some degree to the principal component. The larger a loading's relative magnitude, the more important its presence or absence to the principal component. The third type of information is a score for each cropped image. The localization of each score in the PCA plot depends on the texture features in each image analyzed (18, 30). This function was implemented in the statistical programming environment R (R: A Language and Environment for Statistical Computing, version 3.5.1, 2018, https://www.r-project.org/).

### 2.5. Ethical approval

All applicable international, national, and/or institutional guidelines for the care and use of animals were followed. The study was approved by the Ethics and Administration Committee at the Department of Veterinary Clinical Sciences, Faculty of Health and Medical Sciences, University of Copenhagen (No. 2021-43).

## 3. Results

The study included ultrasound images from 70 healthy male dogs. The youngest dogs in the study population were 7 years of age and the oldest dogs were 14 years. The mean age was 8.9 years (SD = 1.7). The age in full years was distributed as follows (*n*): 7 (14), 8 (22), 9 (12), 10 (10), 11 (7), 12 (2), 13 (1), and 14 (2). The included dog breeds were as follows (number of individuals in parenthesis) : Labrador retriever (5), Field trial cocker spaniel (4), Jack Russell terrier (4), Papillon (4), Dachshund (3), Japanese spitz (3), Shih Tzu (3), and Whippet (3). Nine other breeds ranging is stature from Rottweiler to Chihuahua were represented by two individuals each, and 23 breeds, (again with a wide range in stature) were represented by one individual. The minimum body weight was 2 kg, the maximum was 46 kg and the mean body weight was 17 kg (SD = 12). The BCS was distributed as follows (*n*): 4 (16), 5 (41), 6 (11), and 7 (2). The mean BCS was 5 (SD = 0.71).

Of the 70 dogs, 32 had produced live offspring at some point in their life. The owners of 11 of the 70 dogs reported that the dog had been pharmacologically castrated by treatment with deslorelin acetate at some point in the dog's life. Two dogs were treated within the last 6 months and the remaining dogs had been treated a year and a half ago or earlier. A total of 13 dogs had been treated with osaterone acetate for prostatic hyperplasia. Of these, four dogs were treated within the last year and six dogs were treated two or more years ago. For three dogs, the owner did not know when their dog received treatment.

**Figure 1:**
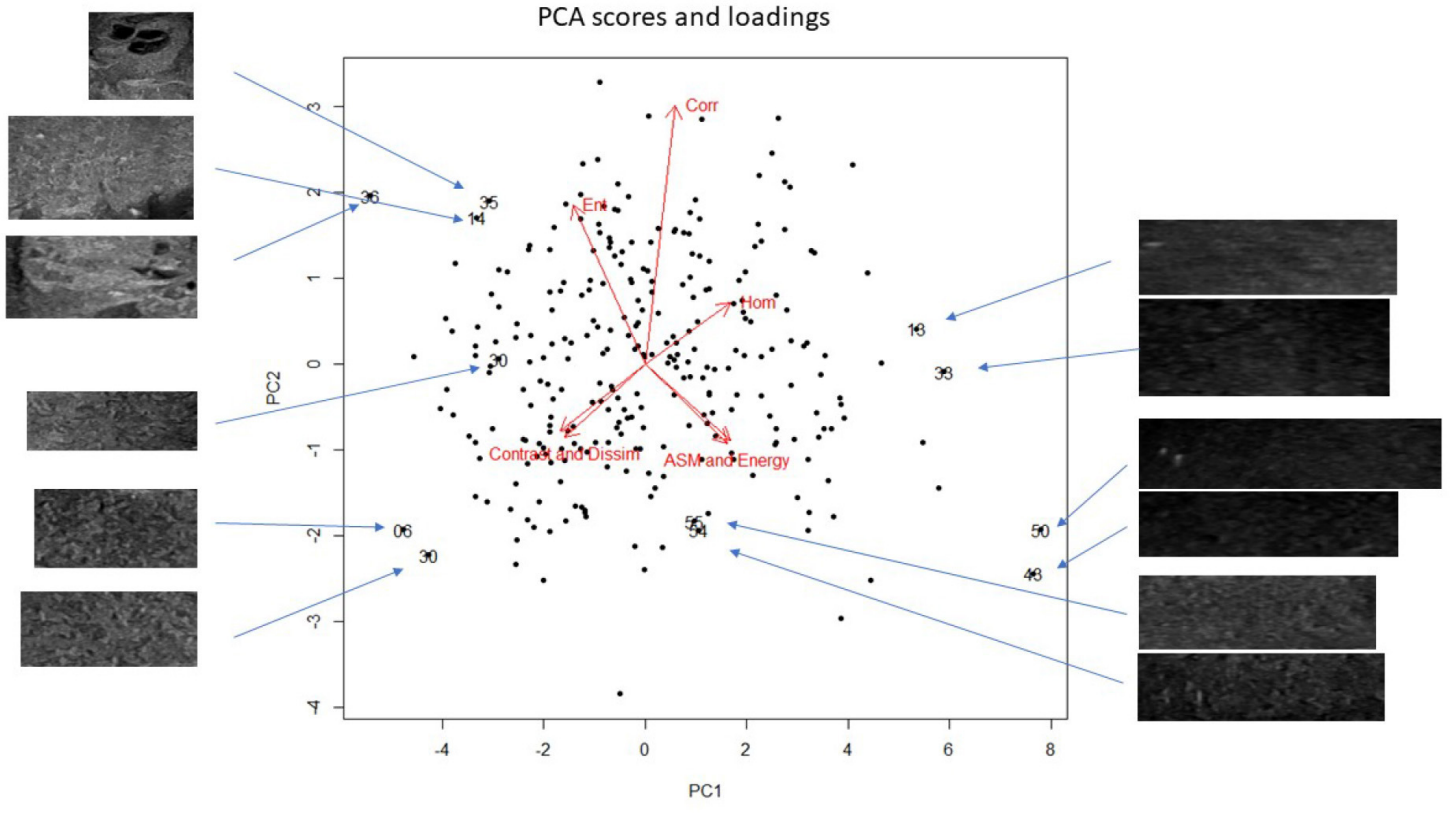
Principal component analysis (PCA) bi-plot showing the scores for each of the cropped regions of interest for all testicular ultrasound images. Each plotting character represents the PCA score for a single cropped image (transverse or sagittal plane). Scores that are close in the plot have similar texture features. The red arrows illustrate the loadings: **(top left)** entropy, **(top middle)** correlation, **(top right)** homogeneity, **(lower left)** contrast and dissimilarity, and **(lower right)** angular second moment and energy. Note that the loadings in the **(lower left)**, dissimilarity and contrast, and **(lower right)**, energy and ASM, almost superimpose, which indicates that these features are highly correlated and have very similar contributions to the PCA. Selected scores are labeled with their unique image number; the associated cropped testicular ultrasound image (signified by an arrow) is shown. This allows a visual comparison of selected images and their PCA scores. The scale of the cropped images has been adjusted to fit the figure, *n* = 280.

All ultrasound examinations were of diagnostic quality. Four images per dog were included in the analysis; a region of interest was selected and cropped manually from one sagittal and one transverse image from the right and left testicles. In total, 280 images were included in the texture analysis and PCA. Of the total variance in our data set, the first principal component (PC1) explained 72.6%, the second principal component explained 21.3%, the third 4.5%, and the fourth to seventh principal components explained the remaining 1.6% (Figure 1). The loading for homogeneity was opposite to that of dissimilarity and contrast, and entropy was opposite energy and ASM. The loadings for contrast and dissimilarity, and for ASM and energy, respectively, are almost superimposed (Figure 1), which indicates that the superimposing features contributed similarly to the model.

**Figure 2:**
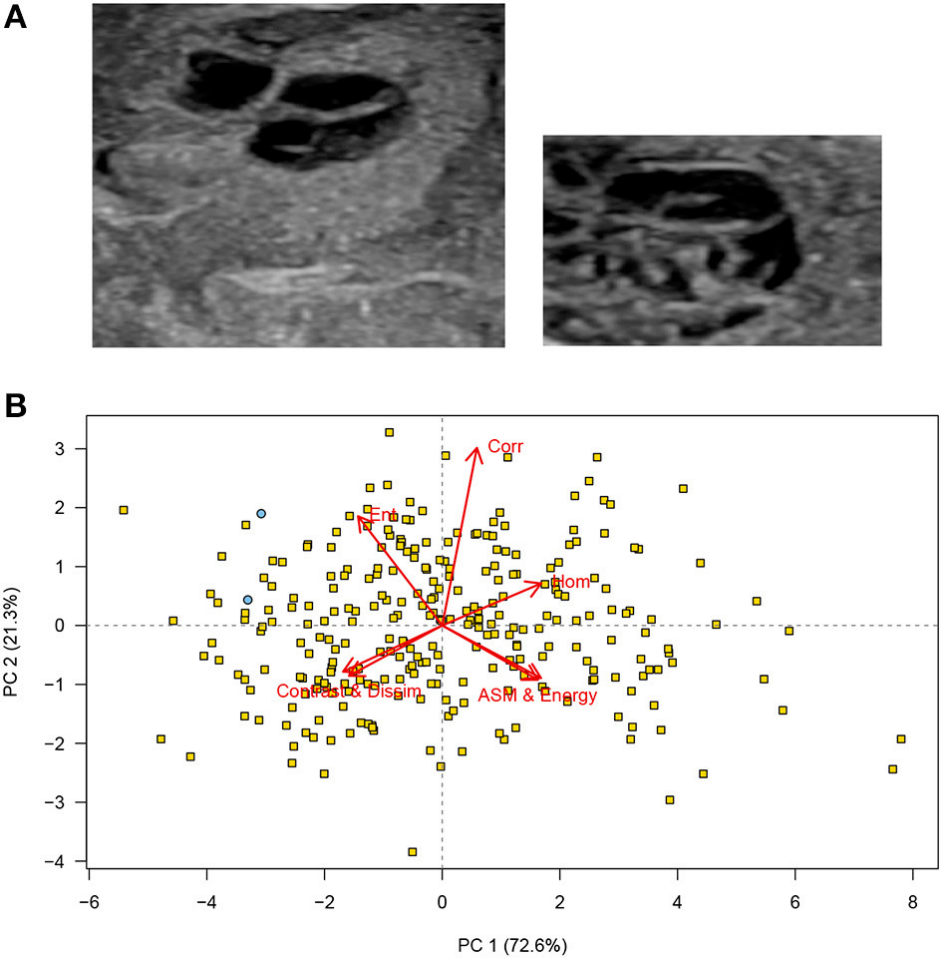
**(A)** Cropped testicular ultrasound images. The scores for these two images are shown as blue circles in the PCA plot. These images were judged to be similar in that both show a septated cystic nodular lesion. **(B)** Principal component analysis (PCA) scores corresponding to the images in **(A)** principal component analysis (PCA) bi-plot showing scores for each of the cropped regions of interest for all testicular ultrasound images. Each plotting character represents the PCA score for a single cropped image (transverse or sagittal plane) and scores that are close in the plot have similar texture features. The red arrows illustrate the loadings: **(top left)** entropy, **(top middle)** correlation, **(top right)** homogeneity, **(lower left)** contrast and dissimilarity, and **(lower right)** angular second moment and energy. The two images shown in **(A)** are identified (blue circles), and all other images are labeled (yellow squares). It can be seen that the two images that contain cysts and appeared similar on subjective, visual assessment are located in close proximity **(upper left)** on the PCA plot, PC, principal component, *n* = 280.

Two images with a roughly similar subjective appearance (cystic septated lesions) were identified among the 280 images (Figure 2A). The scores for both these images were to the left on the first principal component (the x-axis) and on the upper half of the second principal component (y-axis): This confirms that the visual similarity may also be represented in the texture features extracted from the two images (Figure 2B).

**Figure 3:**
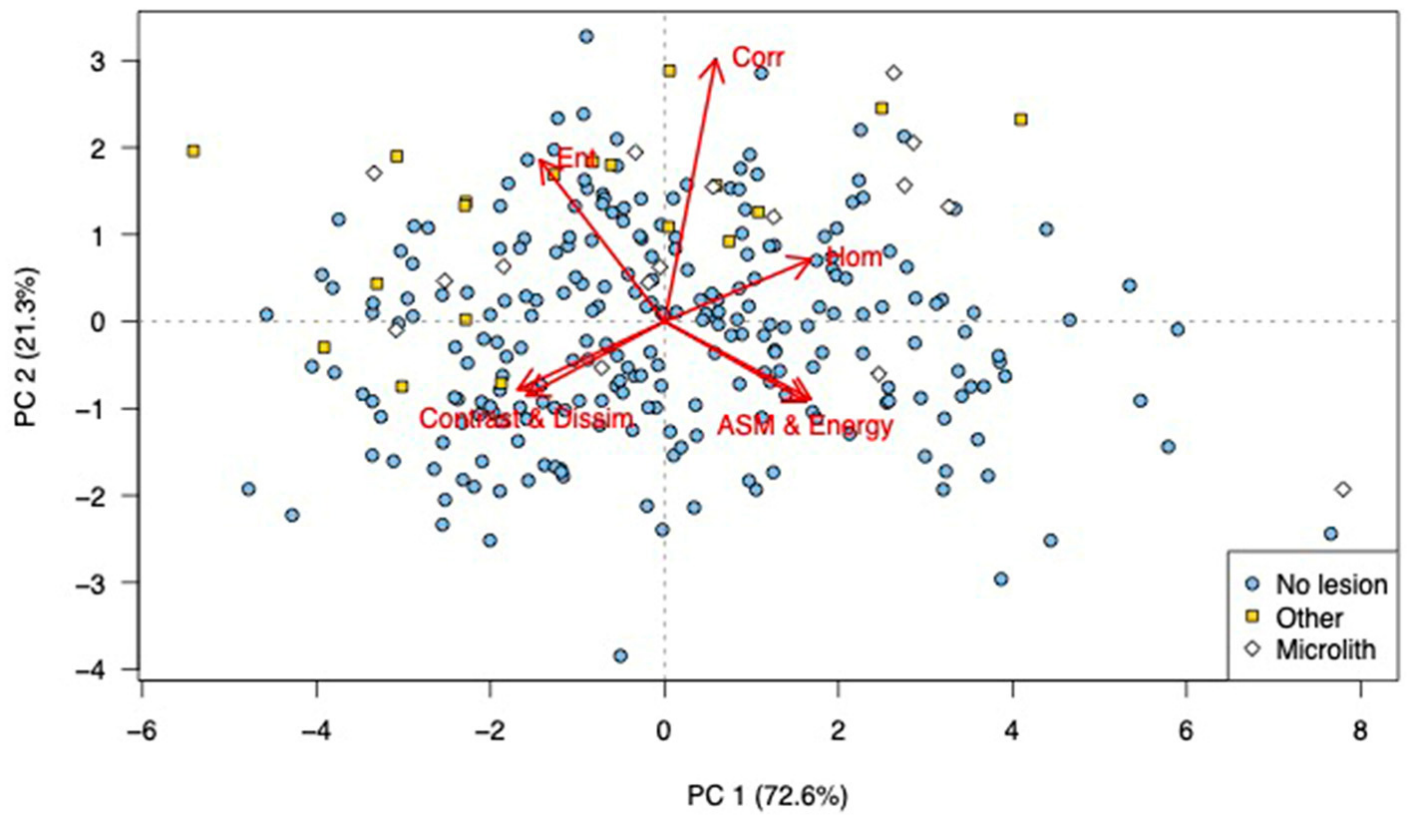
Principal component analysis (PCA) scores labeled to indicate the presence and type of visible abnormalities: PCA bi-plot showing scores for each of the cropped regions of interest for all testicular ultrasound images. Each plotting character represents the PCA score for a single cropped image (transverse or sagittal plane) and scores that are close in the plot have similar texture features. The red arrows illustrate the loadings: **(top left)** entropy, **(top middle)** correlation, **(top right)** homogeneity, **(lower left)** contrast and dissimilarity, and **(lower right)** angular second moment and energy. The scores are labeled according to the subjective human assessment of the cropped image, microliths present (white diamond), another abnormality present (yellow squares), or normal (blue circles). It can be seen that there is a general grouping of both categories of abnormal images, PC, principal component, *n* = 280.

Various abnormal lesions were identified subjectively in 35 of the 280 cropped images. In 16 images, pinpoint-to-small, well-defined, hyperechoic foci were identified without acoustic shadowing. Because of the different appearance of these lesions compared to the rest, these images were classified as containing microliths, although we did not have histopathology to confirm such calcification. This was based on the classification of a similar appearance in men (12, 31, 32). The remaining 19 images with other lesions and areas of non-homogeneous testicular parenchyma were as classified “other.” In the PCA scores plot, most of the images with lesions are to the left and in the upper half of the plot (Figure 3). Thus, the images represented by those scores had higher values for entropy, dissimilarity, and contrast, and lower values for ASM and energy in the first principal component. In the second principal component, values for entropy, correlation, and homogeneity were higher than those for dissimilarity, contrast, energy, and ASM.

**Figure 4:**
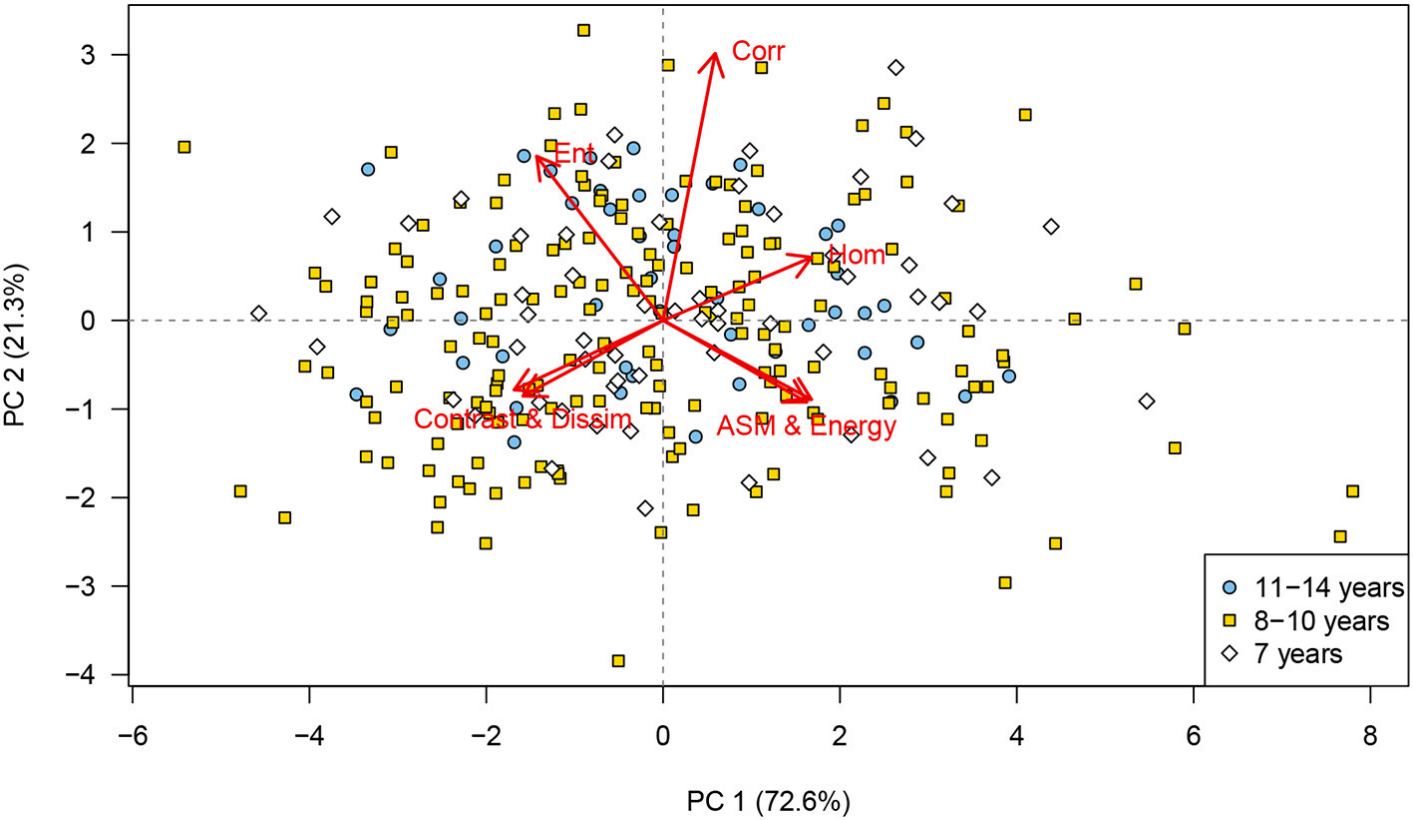
Principal component analysis (PCA) scores labeled according to age group: PCA bi-plot showing scores for each of the cropped regions of interest for all testicular ultrasound images. Each plotting character represents the PCA score for a single cropped image (transverse or sagittal plane) and scores that are close in the plot have similar texture features. The red arrows illustrate the loadings: **(top left)** entropy, **(top middle)** correlation, top right=homogeneity, **(lower left)** contrast and dissimilarity, and **(lower right)** angular second moment and energy. The PCA scores are labeled according to animal age, 7 years (white diamonds), 8–10 years (yellow squares), and 11–14 years (blue circles). It can be seen that there is no identifiable grouping of texture features that relate to animal age, PC, principal component. *n* = 280.

**Figure 5:**
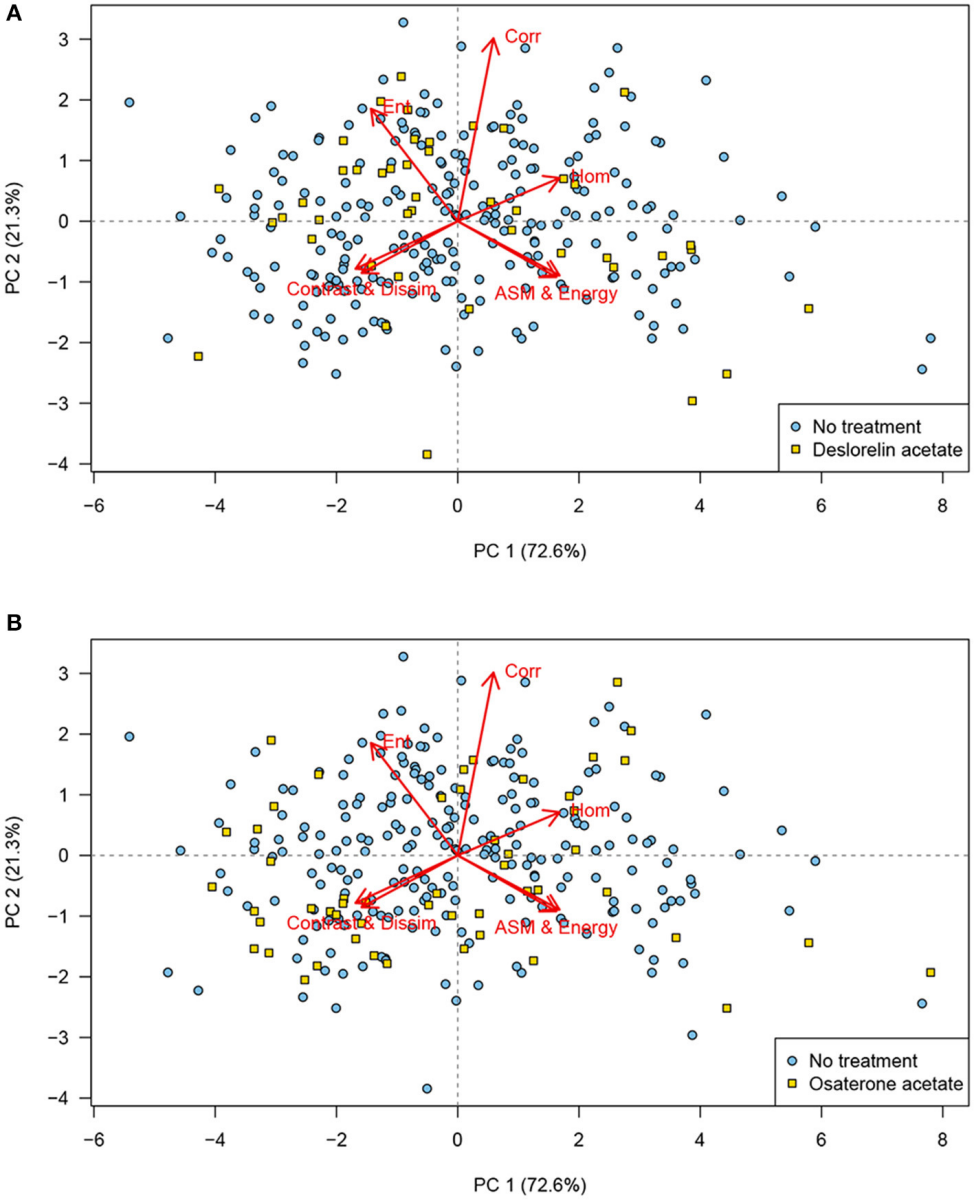
Principal component analysis (PCA) scores labeled according to treatment: PCA bi-plot showing scores for each of the cropped regions of interest for all testicular ultrasound images. Each plotting character represents the PCA score for a single cropped image (transverse or sagittal plane) and scores that are close in the plot have similar texture features. The red arrows illustrate the loadings: **(top left)** entropy, **(top middle)** correlation, **(top right)** homogeneity, **(lower left)** contrast and dissimilarity, and **(lower right)** angular second moment and energy. **(A)** The PCA scores labeled according to treatment with deslorelin acetate at some point in life (yellow squares) have a similar distribution as the scores for dogs not treated with this drug (blue circles). **(B)** The PCA scores labeled according to treatment with osaterone acetate at some point in life (yellow squares) have a similar distribution as the scores for dogs not treated with this drug (blue circles). When comparing the two plots, it can be seen that many of the scores in the osaterone acetate group had a slightly more peripheral distribution than the scores for the deslorelin acetate group, PC, principal component, *n* = 280.

No clustering was identified in the PCA when the scores were labeled according to age group (Figure 4), body condition scores, or the males that had produced offspring. Similarly, the PCA showed no clustering of the scores for dogs treated with deslorelin acetate (Figure 5A) or osaterone acetate (Figure 5B) at some point in life, compared to dogs that had never been treated. However, the PCA showed a slight difference in distribution between the deslorelin acetate group compared to osaterone acetate group. The osaterone acetate group had a slightly more peripheral distribution than the deslorelin acetate group, which indicates that the texture features in the osaterone acetate group were slightly more heterogeneous.

## 4. Discussion

This study demonstrates how texture features from ultrasound images can be extracted and combined with clinical imaging or biological information about individuals, such as the presence of visible lesions in their ultrasound scan (Figure 3), patient age (Figure 4), or previous medical treatment (Figure 5). By use of color coded labeling in the PCA plots the relationship between multiple texture features and other classifiers such as BCS (not shown in our figures) or fertility status (not available in our study) can be examined. Our study also demonstrates how quantitative image features can be explored and compared to subjective identification of similarities and differences in the analyzed images. In this methodology, almost any other quantitative or qualitative parameter with a possible relation to the organ function could be included. Therefore, the use of PCA to study extracted texture features should be of interest in veterinary domains with well-defined disease groups, such as oncology or veterinary reproduction and complex multivariate data.

We could not associate the texture analysis of the ultrasound images in this study with the age. This is in agreement with the results by Moxon et al. (5). We did not necessarily expect age to be a discriminator, since all dogs in our population were at least 7 years. Subjectively, many individuals in our population had more heterogeneous parenchyma than healthy, young male dogs. Had we also included a group of young dogs the result might have been different. Pre-pubertal human individuals have a more hypoechoic ultrasonographic testicular appearance than older individuals and the contrast calculated from GLCM has been reported as significantly different in old individuals compared to younger age groups (33, 34). Similarly, we did not expect the treatments with deslorelin acetate or osaterone acetate to cause data clustering in the analysis because of the long time that passed since most dogs had their treatments. Deslorelin acetate, due to its GnRH agonistic effect, causes atrophy of the seminiferous tubules, resulting in reduced volume and echogenicity of the testes (35, 36). The methodology described in the present study did not take volume into account, and the echogenicity alone should have minimal impact on the texture analysis because the image data were normalized in the analysis. However, the study included very few dogs with an expected ongoing effect of deslorelin acetate and it seems reasonable to assume that any cause of testicular atrophy, or other alterations in the testicular architecture, would also result in altered texture patterns. Osaterone acetate has been reported to affect semen quality transiently for up to 6 weeks post-treatment, but not the subjective appearance of the testes (35, 37).

There was no data clustering when the dogs were labeled according to BCS. The highest BCS in this study was 7 (on a scale up to 9) and only two dogs got this score. More heterogeneous groups are needed to investigate whether BCS can affect the testicular texture in ultrasound images. Similarly, there was no clustering of scores labeled according to the males that had produced offspring. We had no information about semen quality in the included dogs. In this study, the reasons for not producing offspring were more likely related to not having been used for breeding, then to reduced testicular function. In future analyses of ultrasonographic texture and canine fertility, more homogeneous groups and fertility data should be included.

Despite the inclusion of healthy dogs in this study, there were several abnormal testicular lesions in the ultrasound images. Most images with abnormal lesions had higher values for entropy, dissimilarity, contrast, and correlation in the first principal component, whereas energy and ASM contributed less. Entropy, dissimilarity, and contrast have been referred to as edge textures because they result in high values in areas that contain visual and especially irregular edges. This finding is similar to a report of texture analysis of landscapes, where edges are also a characteristic feature in some images (18). For the images where testicular lesions were identified, we did not have histopathology to classify the lesions. However, because PCA is a model based on the data, it illustrates the variance in the chosen features (i.e., texture features) rather than a ground truth classification. To run a supervised machine learning model, however, histopathology or another variable would be needed for classification. From a clinical relevance viewpoint, our results show that there is a wide variety in visual and texture features in dogs considered clinically healthy. Also the finding that certain features are highly correlated (Figure 1) can be used in feature reduction for supervised machine learning applications. Omitting two features e.g., dissimilarity and energy would not impair the model's performance provided their correlated features, contrast and ASM remained. In the machine learning domain the number of training images required is a function of the number of features. Reducing the number of training images is especially important in clinical veterinary medicine where patient numbers are typically small, and in veterinary research applications where the number of research animals is strictly limited.

For a method such as texture analysis to have a diagnostic value, its findings should correlate well with relevant pathophysiology. That is to say, the image appearance of the testicles should correlate to the anatomy and function, or dysfunction, of the testicles. Testicular ultrasonographic texture has been reported to change with impaired semen quality and carcinoma *in situ* (5, 14, 15, 22). Therefore, it is possible that in images with nodular lesions, texture features are altered both because of altered spermatogenesis and because of nodular lesions.

In the diagnosis of testicular disease, other ultrasound methods such as color Doppler or contrast-enhanced ultrasound can be used (6, 9, 38). This study was limited to the analysis of B-mode ultrasound images. It would, however, be possible to include more quantitative and qualitative ultrasound information in the PCA, had that information been available.

There are several limitations to this study. The study population consisted of 70 older dogs (a bias in that only dogs 7 years or older are included), and they were not selected based on differences that would have been expected to reveal specific texture patterns. Thus with respect to age, it is possible that age effects are present in dogs outside the age range included in this study. Had we been able to include a wider age range, the study would have been more generally applicable and assisted in age comparisons. Also, neither histopathology nor information about the semen quality was available. Such information would have been relevant to analyze since a specific pathology may be related to specific texture changes.

This study confirms that objective texture analysis in testicular ultrasound correlates to some of the visual features used in subjective interpretation, such as contrast, uniformity, or edge-like appearances in the images. Texture analysis of testicular ultrasound images can be useful in the search for patterns in the images. In addition, it provides quantitative data for parameters that are highly subjective if assessed by human observers. Therefore, there is a potential for texture analysis data in prediction models in dogs with and without testicular nodules. The PCA analysis indicates that certain features (those that overlap on the PCA bi-plots) are highly and positively correlated. These correlated feature sets are contrast/dissimilarity and ASM/Energy. Future research might take the form of a predictive model. This would require ground truth data and could also include features derived from Doppler and contrast enhanced ultrasound. The predictive model could take the form of a neural network or support vector machine (SVM). The former, if using convolutional neural networks, typically will use images as input data. Large numbers of images (with ground truth) are required and availability of these may be limiting. The latter suggestion, a SVM supervised model, may be of more interest as less images are needed, and because the SVM process being similar to PCA, provides an insight into the relative importance of each image feature in the classification model.

## Data availability statement

The original contributions presented in the study are included in the article/Supplementary material, further inquiries can be directed to the corresponding author.

## Ethics statement

No human studies are presented in the manuscript. The animal studies were approved by Ethics and Administrative Committee, Department of Veterinary Clinical Sciences, University of Copenhagen. The studies were conducted in accordance with the local legislation and institutional requirements. Written informed consent was obtained from the owners for the participation of their animals in this study. No potentially identifiable images or data are presented in this study.

## Author contributions

PH, AM, FM, and PP contributed to the conception and design of the study. TV organized the database. PP, FM, and AM performed the statistical analysis. AM wrote the first draft of the manuscript. PP and FM wrote sections of the manuscript. All authors contributed to the manuscript revision, read, and approved the submitted version.
