# Supplementary figures and images for: A survey of testicular texture in canine ultrasound images
Source: Front Vet Sci. 2023 Aug 11;10:1206916. doi: 10.3389/fvets.2023.1206916 (PMC10450916; doi:10.3389/fvets.2023.1206916)

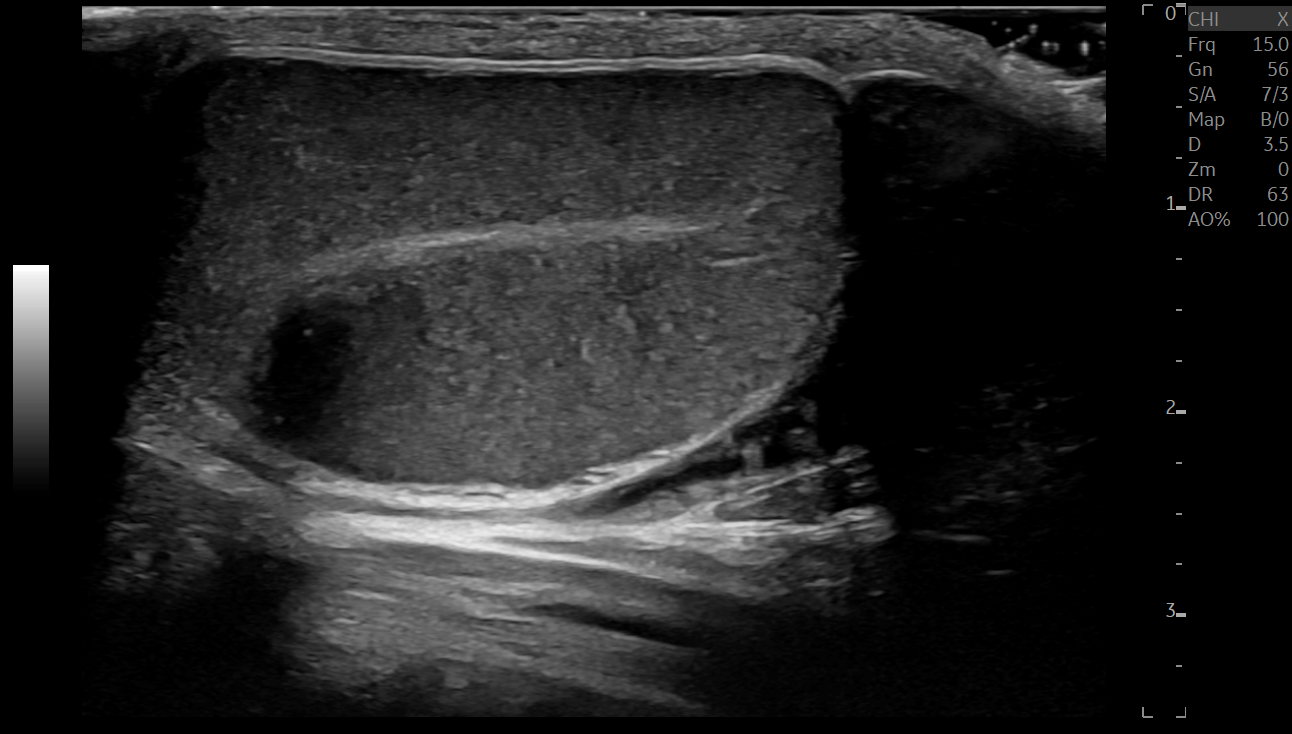

Supplement: Supplementary file 1 [file Data_Sheet_1.zip › Frontiersin----texture-analysis-main/tp_051_ls_0002.png]
